# Positive impact of sodium L-lactate supplementation on blood acid-base status in preterm newborns

**DOI:** 10.1038/s41390-025-03963-9

**Published:** 2025-03-06

**Authors:** Ifrah Omar Ibrahim, Chloé Perrot, Hélène Roumes, Marie-Christine Beauvieux, Olivier Brissaud, Sophie Cramaregeas, Eric Dumas-de-la-Roque, Luc Pellerin, Jean-François Chateil, Olivier Tandonnet, Anne-Karine Bouzier-Sore

**Affiliations:** 1 Univ. Bordeaux, CNRS, CRMSB, UMR 5536, F-33000 Bordeaux, France; 2 Neonatal Intensive Care Unit, Maternity, Bordeaux University Hospital, Bordeaux, France; 3 Biochemistry Department, Bordeaux University Hospital, Bordeaux, France; 4 Neonatal Intensive Care Unit, Children’s Hospital, Bordeaux University Hospital, Bordeaux, France; 5 IRMETIST, U1313, University of Poitiers and CHU of Poitiers, Poitiers, France

## Abstract

**Background:**

Preclinical studies indicate that lactate is a crucial cerebral energy substrate, with Na-L-lactate administration significantly reducing brain lesion volumes and improving motor and cognitive functions following neonatal hypoxia-ischemia in rat pups. Its neuroprotective effects are linked to neuronal metabolic utilization, making it a promising candidate for treating newborns with hypoxia-ischemia encephalopathy, a condition where hypothermia remains the only established therapy. However, before initiating a clinical trial, it is necessary to assess the effects of Na-L-lactate infusion on blood parameters.

**Methods:**

We retrospectively analyzed blood parameters in 60 premature neonates during their first days of life. Among them, 30 received Na-L-lactate instead of Na-Cl to prevent hyperchloremic acidosis. Blood pH, lactatemia, bicarbonates, glycemia, natremia, chloremia, base excess, and hemoglobin were monitored before, during, and after Na-L-lactate infusion.

**Results:**

Our findings showed that Na-L-lactate infusion lowered blood lactate levels while increasing pH from 7.25 to 7.31. After stopping the infusion, lactatemia was 1.9 mM, and pH reached 7.32. Na-L-lactate supplementation effectively restored normal blood pH, maintained natremia, and prevented hyperchloremia. Notably, even in cases of high initial lactatemia, lactate levels decreased during the infusion.

**Conclusion:**

Our data are promising and emphasize the need for further research to explore its potential applications in neonatal clinical care.

**Impact:**

- Sodium L-lactate infusion does not increase blood lactate levels and restores normal pH in premature neonates.
- The study demonstrates that sodium L-lactate infusion avoids hyperchloremia while maintaining sodium levels, offering a potential alternative to sodium chloride.
- These findings highlight the need for additional research studies to further evaluate the safety, efficacy, and potential applications of sodium L-lactate infusion in neonatal care.

## Introduction

Premature birth represents a significant public health challenge.^1^ The incidence of preterm birth is ~9% in Europe and exceeds 13% in Africa,^2^ with survival rates being influenced by the quality of neonatal care provided.^3^ One specific parameter that has undergone scrutiny for its correlation with favorable or unfavorable outcomes is lactatemia, particularly during birth or within the initial 3 h thereafter, with early elevated arterial lactate levels predicting adverse outcomes in preterm neonates.^4^

From a medical perspective, particularly within intensive care units, lactate has historically carried a negative connotation, often regarded as a deleterious waste product and a poor prognostic indicator.^5^ However, advances in our understanding of neuroenergetics increasingly challenge this conventional wisdom. The seminal proposal of an astrocyte-neuron lactate shuttle by Pellerin and Magistretti^6^ fundamentally altered the perception of lactate, a byproduct of carbohydrate metabolism. This model elucidated the critical role of astrocytes, situated at the interface between blood vessels and neurons, in capturing blood glucose and converting it into lactate through glycolysis. Subsequently, this astrocytic lactate is transported to neurons, where it undergoes reconversion into pyruvate, serving as a vital energy substrate through the Krebs cycle and mitochondrial oxidative phosphorylation.

Over the past two decades, accumulating evidence derived from in vitro,^7,8^ ex vivo^9^ and in vivo^10,11^ studies, has consistently highlighted the fact that glucose remains the primary cerebral energy substrate, while emphasizing that lactate is the preferred energy substrate by neurons. This paradigm shift has transformed lactate from being perceived as a harmful metabolite to a cerebral energy substrate that supports neuronal function. Consequently, the notion of lactate as a potential neuroprotective agent has emerged, offering the promise to be used as an alternative substrate in conditions characterized by energy metabolism deficits.

In adults, the neuroprotective benefits of sodium-lactate (Na-Lact) administration are beginning to be documented, particularly in the context of traumatic brain injury.^12–15^ At birth, only two preclinical studies have demonstrated the neuroprotective potential of lactate in the Rice-Vannucci rat model of neonatal hypoxia-ischemia.^16,17^ Evidence shows that lactate metabolism is crucial for neuroprotection, as blocking lactate dehydrogenase—an enzyme that converts lactate into pyruvate and is essential for its metabolism—results in the loss of lactate’s protective effects.^17^ Moreover, the administration of sodium-pyruvate, which is also a hypertonic solution, was not neuroprotective in the Rice-Vannucci model^17^ (although hypertonic solution may be neuroprotective in adults).^18^ Despite these progresses, the correlation between hyperlactatemia and adverse prognosis remains firmly established in pediatric healthcare settings. Interestingly, Ringer’s solution, which contains lactate at a lower concentration, is included in common guidelines for neonatal and pediatric shock, as well as in the ESPGHAN guidelines for parenteral nutrition in neonates and children, although there is no evidence to date of specific benefits or secondary effects in neonates. Ringer’s lactate has been used in neonates with dehydration in Kenya^19^ and in neonatal surgery for decades. However, the use of 1 M Na-lactate solution, with its significantly higher lactate concentration, remains rare, likely due to the negative perception of lactate, and data regarding its administration in newborns are still limited.

The clinical interpretation of elevated blood lactate levels warrants reassessment, as hyperlactatemia may represent an adaptive response to cerebral energy metabolism deficits rather than being inherently deleterious.^20^ To elucidate the potential protective effects of lactate administration in the context of hypoxia-ischemia, it is essential to first investigate the kinetics of lactatemia (and other blood parameters) during the infusion of a sodium-L-lactate solution in a control group. However, as it is not feasible to obtain these data from a cohort of healthy newborns, we conducted a monocentric retrospective clinical study on preterm infants who received sodium-L-lactate solution infusions after birth to correct metabolic acidosis. This study compared the effects of sodium L-lactate solution supplementation with standard parenteral solutions alone on their blood parameters.

## Materials and methods

### Study type

This retrospective single-center descriptive observational study was performed in the neonatal intensive care unit at the maternity of Bordeaux University Hospital. This study was approved by and followed the rules of the Human Ethical Research Committee of Bordeaux Hospital (CHUBX 2023/30). Letters of information and opposition right were sent to all parents.

### Cohort criteria

#### Inclusion criteria

Preterm neonates with gestational age at birth ≥24 weeks, birth weight ≥500 g, metabolic acidosis, non-opposition from holders of parental authority.

#### Exclusion criteria

Systematized neonatal arterial infarction, congenital neurometabolic disease, severe malformations, poor understanding of French among those with parental authority, with no possibility of correct translation.

### Clinical variables

Clinical variables included age, sex, weight at birth, gestational age at birth, lactatemia at birth, Apgar score at 1, 5 and 10 min after birth, origin of prematurity (induced or spontaneous), presence of chorioamnionitis, sepsis, surfactant deficiency, subependymal or intraventricular bleeding, obstructive shock due to pulmonary arterial hypertension, pulmonary hemorrhage, ulcerative enterocolitis, bronchopulmonary dysplasia.

### Biological variables

Measured biological variables included glycemia (mmol/L), lactatemia (mmol/L), blood pH (pH units), natremia (mmol/L), chloremia (mmol/L), and hemoglobin (g/dL). Calculated biological parameters were bicarbonates (mmol/L) and base excess (mEq/L). Biomarkers were analyzed using capillary blood samples on a point-of-care GEM5000 Premier (Werfen, France) that refers to standards. Lactate at birth has been measured in arterial umbilical cord blood, next to birth rooms, on a point-of-care RL1260 device (Siemens Healthiner, France). In all cases, devices have been previously compared to the central lab device with a validation of the result concordance. All devices have been submitted to internal and external quality controls. All medical and paramedical users have been trained and authorized to perform analyses in the application of the normative quality approach (NF ISO 15189).

### Sodium L-lactate infusion

Preterm neonates in the Na-Lact group received intravenous sodium L-lactate infusion (sodium L-lactate AP-HP 11.2% (m/V) solution; sodium = 999 mmol/L = 23 g/L and lactate = 999 mmol/L = 89 g/L). Infusion doses, rates and durations were permanently adapted. Doses ranged from 0.7 mEq/kg/day to 5.4 mEq/kg/day and rates from 0.1 to 0.3 mL/h. Mean time to start the infusion was 1317 ± 245 min after birth and mean time of infusion duration was 110.7 ± 9.7 h.

### Patients

Sixty preterm neonates, 27 girls and 33 boys, born between 24 (1) and 31 (6) weeks (days) of gestation were included. Birth weights ranged from 590 to 1830 g. All babies were on parenteral nutrition (PEDIAVEN NN2, containing no sodium acetate and 0.28 g/L of cystine-chloride). Among this cohort, thirty consecutive preterm infants born between August 2022 to March 2023 received sodium L-lactate infusion (Na-Lact group). The control group was composed of 30 preterm babies who were born before August 2022 (August 2022: starting date of the use of sodium L-lactate to improve the acid-base status and to restore natremia while avoiding hyperchloremia),^21^ in our neonatal intensive care unit. Characteristics of preterm newborns of the cohort are presented in Table 1.

**Table 1:** Summary of some characteristics and parameters of preterm neonates of the cohort.

	Control group	Na-Lact group
Sex (n)	30	30
♀	14 (47%)	13 (43%)
♂	16 (53%)	17 (57%)
Gestational age at birth (week, day/7days a week) [min–max]	27 6/7 ± 3/7* [24 1/7–31 4/7]	26 5/7 ± 2/7* [24 1/7–30 3/7]
Birth weight (g) [min–max]	981.3 ± 53.7* [620–1830]	830.8 ± 35.0* [590–1300]
Apgar 1 min [min–max]	4.1 ± 0.5 [0–9]	4.7 ± 0.4 [1–8]
Apgar 5 min, [min–max]	7.3 ± 0.5 [0–10]	7.2 ± 0.4 [2–10]
Apgar 10 min [min–max]	8.5 ± 0.5 [0–10]	8.4 ± 0.3 [4–10]
Lactatemia at birth (mmol/L) [*n*]	4.6 ± 0.7 [30]	4.1 ± 0.6 [26]
Starting of Na-Lactate 11.2% infusion (min after birth) [min–max]	Ø	1317 ± 245 [60–4860]
Duration of Na-Lactate 11.2% infusion (h) [min–max]	Ø	110.7 ± 9.7 [25–227]

*Apgar* appearance, pulse, grimace, activity, and respiration.

*: *p* < 0.05

Antenatal and medical histories of the cohort are summarized in Table 2. Chorioamnionitis was defined by maternal fever associated with elevated blood CRP in newborns (more or less associated with leukocytosis), with or without bacteriological documentation. Surfactant deficiency was defined by the presence of clinical signs of respiratory distress (based on the Silverman score), decreased oxygen saturation and the presence of radiological signs. Bronchopulmonary dysplasia was defined by an oxygen requirement or the need for ventilatory support beyond 36 weeks of amenorrhea. Obstructive shock due to pulmonary arterial hypertension was defined by the need for treatment with nitric oxide and inotropic support. The classification of necrotizing enterocolitis was based on the modified Bell classification. Intraventricular hemorrhage was detected on transfontanellar ultrasound scans performed several times during hospitalization and graded according to the Papile classification. Sepsis was defined by the presence of suggestive clinical signs associated with at least one positive blood culture between the 4th and 30th day of life. Early bacterial neonatal infections (less than 72 h of life) were not considered. No death occurred during this study.

**Table 2:** Antenatal and medical histories of the cohort.

	Control group (n - %)	Na-Lact group (n - %)
IUGR < 10th percentile	6 - 20%	7 - 23%
Multiple pregnancy	10 - 33%	13 - 43%
Chorioamnionitis	2 - 7%	13 - 43%
Preeclampsia	3 - 10%	3 - 10%
Origin of prematurity		
Induced	14 - 47%	10 - 33%
Spontaneous	16 - 53%	20 - 67%
Antenatal corticotherapy	24 - 80%	24 - 80%
Surfactant deficiency	25 - 83%	25 - 83%
Bronchopulmonary dysplasia	25 - 83%	23 - 77%
Pulmonary hemorrhage	4 - 13%	3 - 10%
Necrotizing enterocolitis	3 - 10%	3 - 10%
Obstructive shock	14 - 47%	14 - 47%
Intraventricular hemorrhage	7 - 23%	14 - 47%
Sepsis	10 - 33%	21 - 70%

*IUGR* intrauterine growth retardation.

### Groups

Preterm neonates in the control group received parenteral nutrition with natremia corrected using chloride saline solution (0.9%; sodium: 154 mmol/L; chloride: 154 mml/L; osmolarity: 308 mOsm/L—control group; *n* = 30). Patients in the Na-Lact group had parenteral nutrition with hypertonic sodium L-lactate solution administration (AP-HP 11.2%; sodium: 999 mmol/L; lactate: 999 mmol/L; osmolarity: 1998 mOsm/L; *n* = 30). To compare biological parameters between the Na-Lact group and the control group (in which preterm neonates did not receive sodium L-lactate infusion), three successive periods were distinguished, based on time windows of the Na-Lact group: (1) time window before sodium L-lactate infusion (0–22 h after birth), (2) time window of sodium L-lactate infusion (22–133 h after birth), and (3) time window after sodium L-lactate infusion (133–377 h after birth). For the control group, means of blood parameter values collected during the same time windows (0–22 h, 22–133 h and 133–377 h) were calculated and compared to the means measured in the Na-Lact group. Thereafter, to analyze deeper the impact of sodium L-lactate infusion on lactatemia, three subgroups in each series were determined: subgroup 1 with blood lactate concentration at birth <2 mmol/L; subgroup 2 with moderate hyperlactatemia at birth (2 ≤ lactatemia ≤ 5 mmol/L) and subgroup 3 with severe hyperlactatemia at birth (blood lactate concentration > 5 mmol/L). Mean times to start the infusion were 1019 ± 370 min, 1863 ± 438 min, and 888 ± 392 min after birth and mean times of infusion duration were 137.4 ± 18.5 h, 95.2 ± 15.3 h, and 104.6 ± 15.2 h, respectively in subgroups 1, 2 and 3. These parameters were also used to calculate means of blood parameter values collected during the corresponding time windows for each subgroup (1, 2, and 3) in the control group. Numbers of patients were 9 in subgroups 1 and 3, and 12 in subgroups 2, in each group.

### Statistics

Data analyses were performed using GraphPad Prism7.00 software. Normal distribution data were expressed as mean ± standard error of the mean (when variables followed a Gaussian distribution) or median ± interquartile range (non-gaussian distribution). Statistical significance of the differences between the two groups was determined using Student’s *t*-test for variables that follow a Gaussian distribution or using a two-tailed Mann-Whitney test for non-gaussian data. For multiple subgroup analyses, statistical comparisons were performed using a One-Way ANOVA followed by Fisher’s LSD post-hoc test for data following a Gaussian distribution, and a Kruskal-Wallis test followed by Dunn’s post-hoc test for data not following a Gaussian distribution. Specifically, non-gaussian data included Chloride, Sodium, and lactatemia in both epochs, as well as pH values in the Na-Lact group, and lactatemia at birth, weight at birth, and Apgar scores. A *p* < 0.05 was considered statistically significant.

## Results

### Cohort

**Fig. 1:**
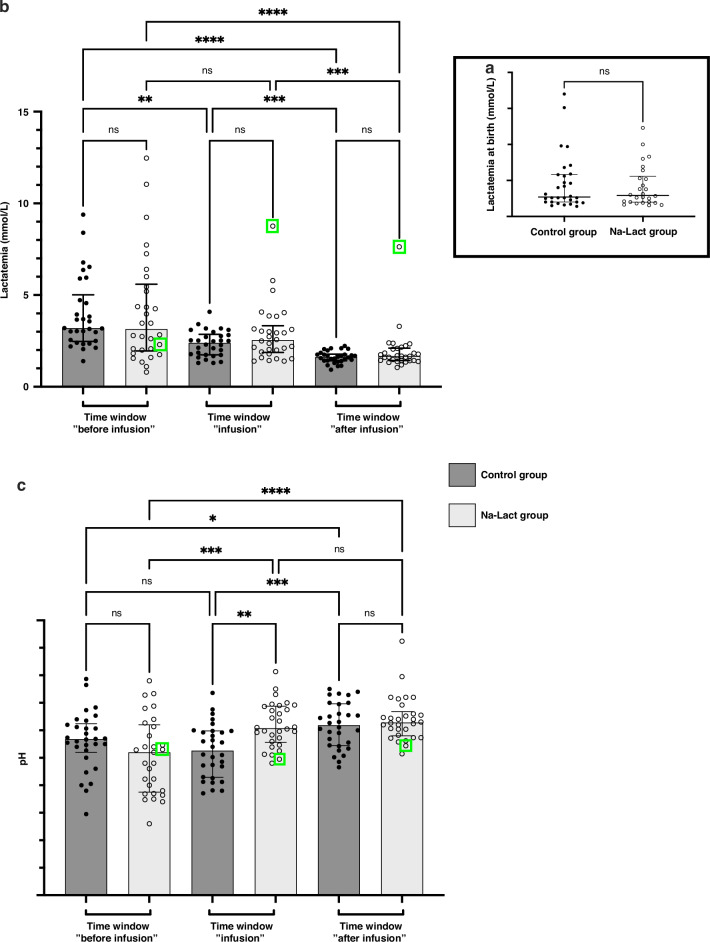
Monitoring of blood lactate concentration and pH. **a** Lactatemia of the cohort at birth. **b** Evolution of lactatemia after birth. Three different time windows after birth were determined: before, during, and after sodium L-lactate infusion. The same time periods after birth were used to analyze the data of the control group. **c** Evolution of blood pH after birth. Same time periods after birth than the one used in (**b**). *: *p* < 0.05, **: *p* < 0.01, ***: *p* < 0.001 and ****: *p* < 0.0001. ns not significant. Green square: data from patient number 29, who presented a major increase in lactatemia during and after the sodium L-lactate infusion, concomitantly with a hemodynamic deterioration in a context of septic shock at 3 ½ days of life. Data are presented as median and interquartile range.

Two groups were considered in this study, the control and the Na-Lact groups. In the Na-Lact group, 30 preterm neonates received infusion of a sodium L-lactate (1 M) solution. This new protocol was set up to administrate sodium while limiting chloride intake, whose increase is deleterious in preterm neonates. Blood parameters were carefully and regularly controlled before, during, and after sodium L-lactate administration. These values were compared with those measured in the control group, which consisted of 30 preterm neonates born before the establishment of this protocol. Gestational ages and weights at birth were slightly smaller in the Na-Lact group compared to the control group. The mean gestational age (in weeks of amenorrhea age) of the newborns in the Na-Lact group was 26 (5) weeks (days) ± 2 days while it was 27 (6) weeks (days) ± 3 days in the control group. Birth weight means were 830 ± 35 g and 981 ± 54 g in Na-Lact and control groups, respectively. However, sex ratio and, more importantly, lactatemia at birth were similar in both groups (Table 1). Blood lactate levels at birth were 4.1 ± 0.6 and 4.6 ± 0.7 mmol/L in Na-Lact and control groups, respectively (Fig. 1a). Apgar (Appearance, Pulse, Grimace, Activity, and Respiration) scores were not statistically different between the two groups, either at 1 min, or at 5 min and nor at 10 min (Table 1). Moreover, except for chorioamnionitis and sepsis, antenatal and clinical courses of patients were similar in the two groups (Table 2). In each group, maternal status was characterized at 80% by antepartum corticosteroid therapy and at 10% by pre-eclampsia, resulting from a dysfunction of the placenta and characterized by high blood pressure and the presence of proteins in urine. At the pulmonary level, in both groups, 83% of newborns were affected by a defect in pulmonary surfactant and secondarily bronchopulmonary dysplasia due to pulmonary immaturity. Ulcerative-necrotizing enterocolitis, an inflammatory digestive disease that mainly affects premature newborns, was present in 10% of preterm newborns in both the Na-Lact and control groups. Patients in the Na-Lact group also had slightly more intraventricular hemorrhages.

### Lactatemia, pH, base excess (BE) and bicarbonates

For each patient in the Na-Lact group, we calculated the mean lactatemia before, during, and after the infusion of an 11.2% sodium L-lactate solution (Fig. 1b). A statistically significant decrease in the mean value of lactatemia was observed while sodium L-lactate was infused (4.1 ± 0.5 mmol/L and 2.9 ± 0.3 mmol/L before and during the infusion, respectively, Fig. 1b and Supplementary Fig. S1A). At the end of the infusion, lactatemia was 1.9 ± 0.2 mmol/L. When compared to the lactatemia measured in the control group during the same time periods after birth, no statistically significant difference was found (Fig. 1b). In the Na-Lact group, no statistically significant differences in lactatemia values were observed between sexes (Supplementary Fig. S1B). Additionally, no impact of birth weight was detected (Supplementary Fig. S1F, G). In subgroup 1, in which preterm infants had a low lactatemia at birth, no difference in lactatemia before, during and after the infusion of a 11.2% sodium L-lactate solution was measured (Supplementary Fig. S1C). A strong increase in blood lactate concentration was measured in only one patient who was the sole preterm infant diagnosed with septic shock at 84 h of life. (Supplementary Fig. S1C, patient 29, green square and Supplementary Fig. S2, patient 29). In subgroup 2 and 3, in which patients had a medium or high lactatemia at birth, a statistically significant decrease in the mean lactatemia values was observed before and during 1 M sodium L-lactate solution infusion (from 3.2 ± 0.3 to 2.3 ± 0.2 mM and from 7.7 ± 0.9 to 3.6 ± 0.5 mM, respectively, Supplementary Fig. S1D, E). All lactatemia values for each patient receiving sodium L-lactate are presented in Supplementary Figs. S2, S3, and S4, corresponding to preterm infants in subgroups 1, 2, and 3, respectively. Kinetics of lactatemia for each patient (excluding patient 29 with sepsis) indicate that blood lactate levels do not exceed the initial lactatemia measured at the start of sodium L-lactate infusion, even in the subgroup 3, in which newborns had high lactatemia at birth (Supplementary Fig. S4). No correlation was observed between the infusion rate of sodium L-lactate and the lactatemia (Supplementary Fig. S5).

Concerning pH, preterm neonates in the control group had blood pH values that were normalized only during the late period after infusion. In contrast, an infusion of sodium L-lactate normalized blood pH during the infusion period itself (Fig. 1c). No statistically significant difference was found between pH values during or after the infusion period in the Na-Lact group (7.31 ± 0.01 and 7.32 ± 0.01, respectively). Supplementary Fig. S6 shows pH values in the different subgroups for the Na-Lact group. In subgroup 3, in which pH values were the lowest in the 1st hours, sodium L-lactate infusion normalized blood pH during the infusion period itself. Bicarbonates and base excess values are presented in Table 3 and in Supplementary Figs. S7 and S8, respectively. No sex difference was observed. Compared to control groups, bicarbonate and base excess levels were normalized faster when preterm neonates received sodium L-lactate (bicarbonate values were 19.5, 22.6 and 25.0 mmol/L in the Na-Lact group compared to 20.4, 19.9 and 24.9 mmol/L in the control group before, during and after infusion time periods, respectively, and base excess values were −7.95, −3.82 and −1.11 mEq/L in the Na-Lact group compared to −6.51, −7.27 and −1.48 mEq/L in the control group before, during and after infusion time periods, respectively).

**Table 3:** Blood biomarkers in both groups

		Time window “before infusion”	Time window “infusion”	Time window “after infusion”
Lactate (mmol/L)	**Control Group**	**4.0 ± 0.4**^**†††**^	**2.3 ± 0.1**	**1.6 ± 0.1**
	Subgoup 1	2.4 ± 0.2	2.2 ± 0.2†	1.5 ± 0.1
	Subgroup 2	3.6 ± 0.3^††††^	1.9 ± 0.1	1.7 ± 0.1
	Subgroup 3	6.0 ± 0.7^†††^	3.1 ± 0.2	1.6 ± 0.1
	**Na-Lact Group**	**4.1 ± 0.5**^**††**^	**2.9 ± 0.3**^**†**^	**1.9 ± 0.2**
	Subgoup 1	1.6 ± 0.2*^††^	2.3 ± 0.2†	1.7 ± 0.2
	Subgroup 2	3.2 ± 0.3††	2.3 ± 0.2	1.7 ± 0.1
	Subgroup 3	7.7 ± 0.9* ^††††^	3.6 ± 0.5†	1.9 ± 0.1
pH (pH units)	**Control Group**	**7.28 ± 0.01**	**7.26 ± 0.01**^**†††**^	**7.31 ± 0.01**
	Subgoup 1	7.27 ± 0.02	7.26 ± 0.01†	7.31 ± 0.01
	Subgroup 2	7.31 ± 0.01†	7.27 ± 0.02†	7.32 ± 0.01
	Subgroup 3	7.25 ± 0.02	7.26 ± 0.02	7.30 ± 0.01
	**Na-Lact Group**	7.25 ± 0.01*^††††^	7.31 ± 0.01***	**7.32 ± 0.01**
	Subgoup 1	7.28 ± 0.03	7.32 ± 0.01*	7.32 ± 0.01
	Subgroup 2	7.27 ± 0.02*^††^	7.31 ± 0.01*	7.32 ± 0.01
	Subgroup 3	7.21 ± 0.02^†††^	7.30 ± 0.01	7.32 ± 0.02
Base excess (mEq/L)	**Control Group**	**−6.51 ± 0.45**	**−7.27 ± 0.61**^**††††**^	**−1.48 ± 0.56**
	Subgoup 1	−5.43 ± 0.61	−6.98 ± 0.97^††††^	−1.33 ± 1.13
	Subgroup 2	−6.19 ± 0.71	−6.83 ± 1.19^††††^	−1.34 ± 0.92
	Subgroup 3	−8.03 ± 0.84	−8.15 ± 0.95^†††^	−1.80 ± 0.97
	**Na-Lact Group**	**−7.95 ± 0.95**^**††††**^	−3.82 ± 0.35****^†††^	**−1.11 ± 0.44**
	Subgoup 1	6.18 ± 1.44	−3.80 ± 0.78*^††^	−1.31 ± 0.49
	Subgroup 2	−6.89 ± 1.39†	−3.63 ± 0.51*^††^	−1.00 ± 0.64
	Subgroup 3	−9.97 ± 1.88^†††^	−4.03 ± 0.66††	−1.25 ± 1.21*
Bicarbonates (mmol/L)	**Control Group**	**20.4 ± 0.4**	**19.9 ± 0.5**^**††††**^	**24.9 ± 0.5**
	Subgoup 1	21.7 ± 0.6	20.2 ± 0.9^††††^	25.0 ± 1.0
	Subgroup 2	20.2 ± 0.6	20.2 ± 1.0^†††^	24.9 ± 0.8
	Subgroup 3	19.3 ± 0.8	19.1 ± 0.7^†††^	24.7 ± 0.9
	**Na-Lact Group**	**19.5 ± 0.9**^**††††**^	22.6 ± 0.4***^†††^	**25.0 ± 0.4**
	Subgoup 1	20.7 ± 1.4	22.6 ± 0.8*	24.7 ± 0.5
	Subgroup 2	20.3 ± 1.2†	22.7 ± 0.5*^††^	25.3 ± 0.6
	Subgroup 3	17.8 ± 1.9††	22.5 ± 0.7*	25.1 ± 1.1
Sodium (mmol/L)	**Control Group**	**129.5 ± 0.9**^**††††**^	**135.7 ± 0.7**	**135.5 ± 0.5**
	Subgoup 1	127.2 ± 1.5^††††^	135.8 ± 1.0	135.6 ± 0.5
	Subgroup 2	131.7 ± 1.1^†††^	136.6 ± 1.0	136.0 ± 0.6
	Subgroup 3	128.7 ± 2.0††	134.3 ± 1.4	134.6 ± 1.1
	**Na-Lact Group**	**128.9 ± 0.7**^**††††**^	133.8 ± 0.6*	133.0 ± 0.7*
	Subgoup 1	129.9 ± 2.3†	134.5 ± 0.7	131.8 ± 1.6*
	Subgroup 2	129.5 ± 1.0††	134.1 ± 1.0	133.2 ± 1.0*
	Subgroup 3	127.4 ± 1.2†	132.6 ± 1.2	133.9 ± 1.0
Chloride (mmol/L)	**Control Group**	**101.0 ± 0.7**^**††††**^	**106.1 ± 0.8**^**†**^	**103.3 ± 0.7**
	Subgoup 1	100.0 ± 1.4††	106.1 ± 1.2	103.1 ± 1.0
	Subgroup 2	102.7 ± 1.0†	106.6 ± 1.3	104.2 ± 0.8
	Subgroup 3	99.9 ± 1.4††	105.5 ± 1.6	102.3 ± 1.9
	Na-Lact Group	99.7 ± 0.7†	102.3 ± 0.6***^††^	99.8 ± 1.1**
	Subgoup 1	101.5 ± 1.8	103.0 ± 1.0†	98.1 ± 2.6*
	Subgroup 2	101.4 ± 0.7	102.7 ± 1.0*	100.4 ± 1.5*
	Subgroup 3	96.5 ± 0.6†	101.0 ± 1.1*^††^	100.8 ± 1.7

^†^Significantly different from the measured value in the following time period. *Significantly different between the Na-Lact and the control groups. ^††^ or *: *p* < 0.05; ^††^ or **: *p* < 0.01; ^††^ or ***: *p* < 0.001; ^††††^ or ****: *p* < 0.0001.

### Natremia and chloremia

**Fig. 2:**
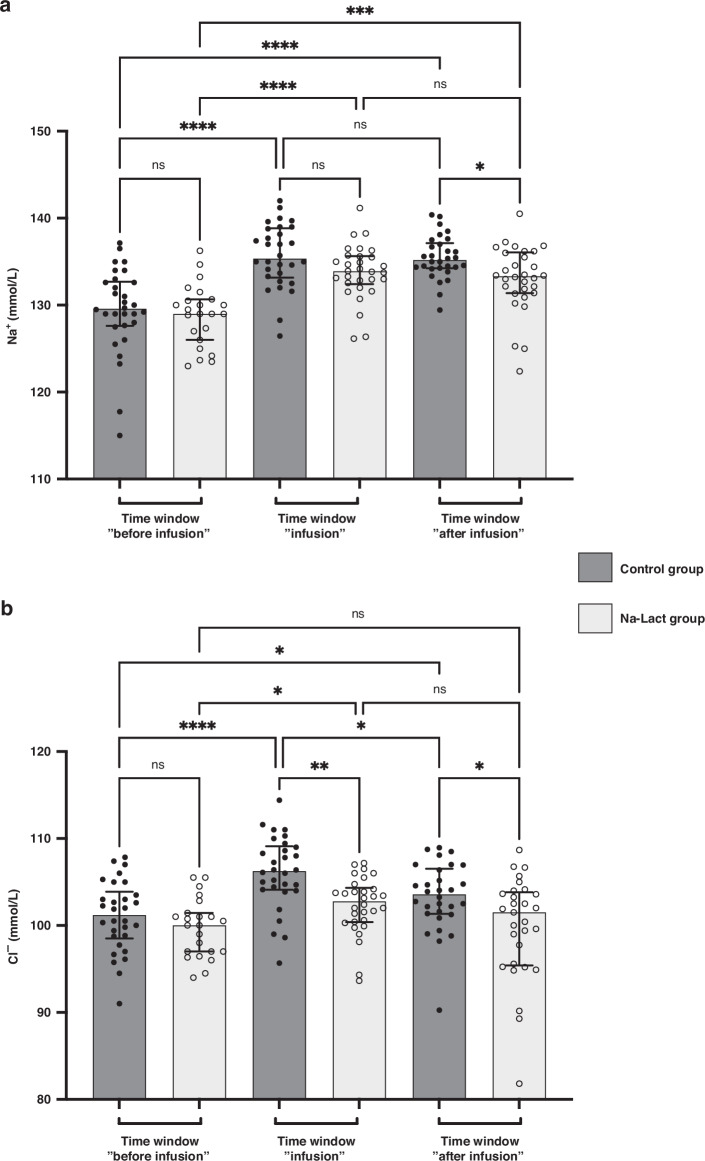
Monitoring of blood sodium and chloride concentrations. **a** Evolution of natremia after birth. Three different time windows after birth were determined: before, during, and after sodium L-lactate infusion. The same time periods after birth were used to analyze the data of the control group. **b** Evolution of chloremia after birth (same time periods after birth than the one used in **a**). *Significantly different. *: *p* < 0.05, **: *p* < 0.01, ***: *p* < 0.001 and ****: *p* < 0.0001. ns: not significant. Data are presented as median and interquartile range.

Natremia and chloremia values before, during, and after infusion time periods are presented in Fig. 2a, b, respectively, and in Table 3. In both groups, an increase in blood sodium levels was measured during the time window “infusion”. Subsequently, these values were upheld during the time window “after infusion”, in both groups. However, during and following the sodium L-lactate infusion periods, natremia was slightly lower in the Na-Lact group compared to values in the control group (133.8 and 133.0 mmol/L compared to 135.7 and 135.5 mmol/L, respectively).

Concerning chloremia, in the Na-Lact group, values were 99.7, 102.3, and 99.8 mmol/L before, during, and after sodium L-lactate infusion whereas values were 101.0, 106.1, and 103.3 mmol/L during the corresponding time periods in the control group.

Finally, the anion gap was calculated and was >12 only in the Na-Lact Subgroup 3, before perfusion of lactate (13.1 mEq/l). During and after Na-lactate perfusion, anion gap values were 9.1 and 8 mEq/l, respectively.

## Discussion

Pre-clinical studies have demonstrated that lactate is an important cerebral energy substrate and that administering sodium L-lactate solution significantly reduces brain lesion volumes and restores motor and cognitive functions following neonatal hypoxia-ischemia in rat pups (Rice-Vannucci model).^10,16,17,22^ However, to elucidate the potential protective effects of lactate administration in the context of neonatal hypoxia-ischemia, it is necessary to first examine the kinetics of lactatemia and other blood parameters during the infusion of a sodium L-lactate solution in a control group. Since it is not feasible to obtain these data from a cohort of healthy newborns, we examined a set of blood parameters in 60 premature neonates during their 1st days of life, with 30 receiving standard parenteral nutrition (nutritional management of preterm infants is a major challenge for neonatologists)^23^ containing chloride saline solution and the remaining 30 receiving sodium L-lactate infusion as an alternative to sodium chloride to prevent detrimental hyperchloremic acidosis.

Birth initiates significant alterations in water and electrolyte levels. In preterm infants, disruptions of extracellular fluid and electrolyte balance are commonly observed, primarily due to the underdeveloped distal nephron, resulting in a diminished capacity to concentrate urine.^24^ Failure to manage water loss adequately places premature infants at risk of volume depletion and hypernatremia.^25^ On the other hand, the immaturity of the proximal renal tubule in preterm infants can cause the decreased reabsorption of sodium and therefore hyponatremia.^26^ Consequently, the management of fluid and electrolyte levels constitutes a crucial aspect of supportive care for preterm infants, typically administered through parenteral infusion. However, caution is warranted regarding the infusion of normal saline solution in premature infants, which contains equal concentrations of sodium and chloride. While chloride requirements are deemed equivalent to sodium, the urinary excretion of chloride may be less efficient than that of sodium, thereby disrupting the acid-base balance and precipitating hyperchloremia-associated metabolic acidosis.^27,28^ Moreover, fluctuations in blood sodium levels have been correlated with increased morbidity and mortality rates among preterm infants, including incidents of intraventricular hemorrhage.^29–31^ Given these considerations, the potential therapeutic benefits of sodium L-lactate infusion for restoring fluid and electrolyte balance in preterm newborns have been investigated, comparing its characteristics with those of standard saline solution. While the management of natremia was comparable between the two groups, the use of sodium L-lactate avoided the increase in chloremia measured in the control groups, in which preterm newborns received sodium chloride in their parenteral nutrition. Since excess chloride is one reason for metabolic acidosis in neonates,^21^ replacing a portion of chloride with lactate in total parenteral nutrition has a positive impact on the blood parameters of preterm infants.

In our study, the perinatal clinical profiles of the cohort showed similarity between the two groups and were consistent with the prematurity state of newborns, encompassing conditions such as pre-eclampsia, intrauterine growth retardation, ulcerative necrotizing enterocolitis, surfactant deficiency, pulmonary hemorrhage, and bronchopulmonary dysplasia. Apgar scores at birth were initially low but rapidly surpassed 7 in both groups. Sex represents a significant variable in preterm outcomes, with male infants generally exhibiting poorer outcomes compared to females.^32^ While the impact of sex on susceptibility to prematurity remains a topic of debate,^33^ sampling of participants was sex-balanced in our study thus limiting bias. Moreover, data analysis was conducted in a sex-balanced manner, revealing no significant differences in any parameters concerning sex. Given that a primary objective of this study was to monitor the kinetics of lactatemia during sodium L-lactate infusion, we carefully examined lactatemia levels at birth in our cohort, finding them to be similar between the two groups. Hyperlactatemia at birth, defined as blood lactate levels exceeding 2 mmol/l, was evident in 70% of the preterm infants in both groups. Among these, 30% exhibited moderate hyperlactatemia (blood lactate concentration between 2 and 5 mmol/L), and 40% exhibited severe hyperlactatemia (blood lactate concentration >5 mmol/L). Subsequently, the impact of sodium L-lactate infusion on lactatemia and blood pH was meticulously monitored.

When comparing the Na-Lact and control groups, sodium L-lactate infusion facilitated the normalization of pH levels, along with enhancements in bicarbonate levels and base excess. When sodium lactate is infused into the body, it is metabolized into bicarbonate, an alkaline buffer that neutralizes excess acidity in the blood.^34^ In contrast, the standard saline solution was associated with a delay in pH normalization compared to sodium L-lactate. The sodium chloride solution contains 154 mEq/l of sodium and chloride. In contrast, the sodium L-lactate 11.2% solution contains 999 mEq/l of sodium and lactate. This compound exerts an alkalinizing effect through the metabolism of lactate, which in turn reduces water dissociation and proton concentration.^34^ The improvement in blood bicarbonate levels following sodium L-lactate infusion contributed to pH elevation. Notably, bicarbonates in plasma and hemoglobin in erythrocytes constitute the two primary fetal and newborn buffering systems, each contributing ~35% to the total buffering capacity.^35^ Carbon dioxide, a byproduct of the Krebs cycle (which can be fueled by lactate following its conversion to pyruvate), diffuses across cell membranes into the blood. It is subsequently absorbed by erythrocytes and converted to H_2_CO_3_ and HCO_3_^−^, thereby buffering excess protons through blood bicarbonates. Base excess, a concept introduced by Siggaard-Andersen in 1960, serves as a respiratory-independent marker of metabolic acidosis/alkalosis and as an indicator of disorder severity.^36,37^ Values of base excess ranging from -2 to 2 mmol/L are considered normal. Analyses indicated that the control group exhibited greater acidosis in the initial hours, with lower pH and base excess compared to the Na-Lact group (pH of 7.26 vs. 7.31 and base excess of −8.3 vs. −3.8).

Alternative infusion solutions have been explored in premature infants to circumvent chloride elevation, which contributes to metabolic acidosis. Sodium bicarbonate infusion, though sparingly recommended due to adverse effects and limited efficacy evidence, has been utilized in select cases.^38^ Sodium acetate has also been employed for prevention of hyperchloremia^39–41^ and promote bicarbonate metabolism, addressing metabolic acidosis in preterm infants.^40,42,43^ The use of sodium acetate in parenteral nutrition is considered standard of care, at least in the UK according to the NICE guidelines. In a randomized controlled trial, Peters et al. demonstrated that the chloride group exhibited greater acidosis with lower pH and base excess than the acetate group on day 5 of life (pH of 7.30 vs. 7.35 and BE of −5.7 vs. −2.1, respectively).^43^ Therefore, similar conclusions to ours were drawn when comparing the use of sodium chloride in the parenteral nutrition to sodium acetate infusion. However, compared to these alternatives, sodium L-lactate presents distinct advantages. The therapeutic efficacy of sodium L-lactate has been substantiated across various clinical scenarios. Its utility has been demonstrated in the management of septic shock, wherein hyperosmolar sodium L-lactate resuscitation exhibited notable lactate clearance in severe pediatric sepsis (intravenous bolus of 5 ml/kg, 10 min).^44^ Additionally, sodium L-lactate has proven to be beneficial in cardiac post-surgery settings (intravenous infusion 2.5 ml/kg, 15 min)^45^ and traumatic brain injury cases (1.5 ml/kg, 15 min).^46^ Moreover, preterm infants are prone to white matter brain injury, characterized by gliosis and white matter foci activation involving astrocytes and microglia.^47,48^ Glutamate excitotoxicity, a prominent feature in preterm newborn neuropathology, is implicated in this context.^48^ Preliminary investigations in traumatic brain injury settings have indicated a favorable impact of hypertonic lactate on reducing both excitotoxicity and intracranial pressure.^14^ Finally, one major advantage to use sodium L-lactate relies on the potential role of lactate to serve as a cerebral energy substrate^6,11,49,50^ and therefore as a potential neuroprotective agent.^16,17,22,51,52^ Exogenous lactate supply through sodium L-lactate infusion may serve as a preferential energy substrate for the premature brain, preserving cerebral glucose for the pentose phosphate pathway and enhancing the brain’s redox equilibrium by sustaining glutathione levels. The contribution of sodium L-lactate infusion to cerebral energy metabolism maintenance and its role in oxidative stress regulation merit further investigation in prospective clinical studies.

It is important to highlight that this study has several strengths. Notably, it is the first to demonstrate that a slow-rate infusion of a 1 M sodium L-lactate solution does not lead to an increase in lactatemia in preterm infants, providing valuable insights into its physiological effects in this vulnerable population. Additionally, it provides the first detailed description of trends in electrolytes during such infusions. However, the study is not without limitations. One significant limitation is that it is an observational study with a small sample size. In this study, one infant experienced an increase in serum lactate levels during the infusion. However, this was attributed to septic shock. Despite this complication, the infant recovered well, which is why we decided not to remove this patient from the analyzed cohort. Moreover, since not all infants underwent brain MRI studies, such data were not included, representing a limitation, especially given the emphasis on the potential effects of sodium lactate on the brain. These factors collectively underscore the need for larger, controlled studies with comprehensive data collection to validate and expand upon the findings presented here.

## Conclusion

The results of this retrospective clinical study indicate that, for premature infants, the infusion of sodium L-lactate does not lead to an increase in blood lactate levels, even in cases in which lactatemia was elevated at birth, while maintaining normal sodium levels. Instead, it facilitates a more rapid normalization of pH and prevents an increase in chloride levels. Furthermore, given the beneficial effects of lactate on brain metabolism, these findings underscore the need for further research. Preclinical and clinical studies are essential to evaluate the safety, efficacy, and potential applications of sodium L-lactate infusion in neonatal care, particularly in the context of neonatal hypoxia-ischemia. Such studies should include comparisons between hypertonic sodium lactate, Ringer’s solution, and sodium acetate, as well as the incorporation of brain MRI examinations, among other approaches.

## Supplementary information

PedRes Suppl Fig

## Data Availability

The datasets generated during and/or analyzed during the current study are available from the corresponding author upon reasonable request.

## References

[1] Vogel, J. P. et al. The global epidemiology of preterm birth. *Best. Pract. Res. Clin. Obstet. Gynaecol.* **52**, 3–12 (2018). 29779863 10.1016/j.bpobgyn.2018.04.003

[2] Chawanpaiboon, S. et al. Global, regional, and national estimates of levels of preterm birth in 2014: a systematic review and modelling analysis. *Lancet Glob. Health* **7**, e37–e46 (2019). 30389451 10.1016/S2214-109X(18)30451-0 PMC6293055

[3] Symington, A. & Pinelli, J. Developmental care for promoting development and preventing morbidity in preterm infants. *Cochrane Database Syst. Rev.* **2006**, CD001814 (2006). 16625548 10.1002/14651858.CD001814.pub2 PMC8962209

[4] Groenendaal, F., Lindemans, C., Uiterwaal, C. S. & de Vries, L. S. Early arterial lactate and prediction of outcome in preterm neonates admitted to a neonatal intensive care unit. *Biol. Neonate* **83**, 171–176 (2003). 12660433 10.1159/000068927

[5] Zhu, W. et al. Proton magnetic resonance spectroscopy in neonates with hypoxic-ischemic injury and its prognostic value. *Transl. Res.* **152**, 225–232 (2008). 19010293 10.1016/j.trsl.2008.09.004

[6] Pellerin, L. & Magistretti, P. J. Glutamate uptake into astrocytes stimulates aerobic glycolysis: a mechanism coupling neuronal activity to glucose utilization. *Proc. Natl Acad. Sci. USA* **91**, 10625–10629 (1994). 7938003 10.1073/pnas.91.22.10625 PMC45074

[7] Bouzier-Sore, A. K., Voisin, P., Canioni, P., Magistretti, P. J. & Pellerin, L. Lactate is a preferential oxidative energy substrate over glucose for neurons in culture. *J. Cereb. Blood Flow. Metab.* **23**, 1298–1306 (2003). 14600437 10.1097/01.WCB.0000091761.61714.25

[8] Bouzier-Sore, A. K. et al. Competition between glucose and lactate as oxidative energy substrates in both neurons and astrocytes: a comparative NMR study. *Eur. J. Neurosci.* **24**, 1687–1694 (2006). 17004932 10.1111/j.1460-9568.2006.05056.x

[9] Bouzier, A. K. et al. The metabolism of [3-(13)C] lactate in the rat brain is specific of a pyruvate carboxylase-deprived compartment. *J. Neurochem.* **75**, 480–486 (2000). 10899922 10.1046/j.1471-4159.2000.0750480.x

[10] Roumes, H. et al. Lactate transporters in the rat barrel cortex sustain whisker-dependent bold fMRI signal and behavioral performance. *Proc. Natl. Acad. Sci. USA* **118**, e2112466118 (2021). 10.1073/pnas.2112466118 PMC8617497 34782470

[11] Roumes, H., Pellerin, L. & Bouzier-Sore, A. K. Astrocytes as metabolic suppliers to support neuronal activity and brain functions. *Essays Biochem.* **67**, 27–37 (2023). 36504117 10.1042/EBC20220080 PMC10011397

[12] Patet, C. et al. Neuroenergetic response to prolonged cerebral glucose depletion after severe brain injury and the role of lactate. *J. Neurotrauma* **32**, 1560–1566 (2015). 25790152 10.1089/neu.2014.3781

[13] Quintard, H. et al. Improvement of neuroenergetics by hypertonic lactate therapy in patients with traumatic brain injury is dependent on baseline cerebral lactate/pyruvate ratio. *J. Neurotrauma* **33**, 681–687 (2016). 26421521 10.1089/neu.2015.4057 PMC4827289

[14] Bouzat, P. et al. Cerebral metabolic effects of exogenous lactate supplementation on the injured human brain. *Intensive Care Med.* **40**, 412–421 (2014). 24477453 10.1007/s00134-013-3203-6

[15] Millet, A. et al. Hypertonic sodium lactate reverses brain oxygenation and metabolism dysfunction after traumatic brain injury. *Br. J. Anaesth.* **120**, 1295–1303 (2018). 29793596 10.1016/j.bja.2018.01.025

[16] Tassinari, I. D. & de Fraga, L. S. Potential use of lactate for the treatment of neonatal hypoxic-ischemic encephalopathy. *Neural Regen. Res.* **17**, 788–790 (2022). 34472472 10.4103/1673-5374.322459 PMC8530120

[17] Roumes, H. et al. Neuroprotective role of lactate in rat neonatal hypoxia-ischemia. *J. Cereb. Blood Flow. Metab.* **41**, 342–358 (2021). 32208801 10.1177/0271678X20908355 PMC7812521

[18] Cook, A. M. et al. Guidelines for the acute treatment of cerebral edema in neurocritical care patients. *Neurocrit Care* **32**, 647–666 (2020). 32227294 10.1007/s12028-020-00959-7 PMC7272487

[19] Akech, S. et al. The prevalence and management of dehydration amongst neonatal admissions to general paediatric wards in Kenya-a Clinical Audit. *J. Trop. Pediatr.* **64**, 516–522 (2018). 29329448 10.1093/tropej/fmx108 PMC6276025

[20] Marikar, D., Babu, P. & Fine-Goulden, M. How to interpret lactate. *Arch. Dis. Child Educ. Pract.* **106**, 167–171 (2021). 10.1136/archdischild-2020-319601 32887681

[21] Groh-Wargo, S., Ciaccia, A. & Moore, J. Neonatal metabolic acidosis: effect of chloride from normal saline flushes. *JPEN J. Parenter. Enter. Nutr.* **12**, 159–161 (1988). 10.1177/0148607188012002159 3129591

[22] Tassinari, I. D. et al. Lactate administration reduces brain injury and ameliorates behavioral outcomes following neonatal hypoxia-ischemia. *Neuroscience* **448**, 191–205 (2020). 32905840 10.1016/j.neuroscience.2020.09.006

[23] Iacobelli, S., Lapillonne, A., Boubred, F. & members, E. N. C. Early postnatal nutrition and renal consequences in preterm infants. *Pediatr. Res.*10.1038/s41390-024-03080-z. Online ahead of print (2024). 10.1038/s41390-024-03080-z 38374220

[24] Speller, A. M. & Moffat, D. B. Tubulo-vascular relationships in the developing kidney. *J. Anat.* **123**, 487–500 (1977). 858697 PMC1234546

[25] Iacobelli, S. & Guignard, J. P. Maturation of glomerular filtration rate in neonates and infants: an overview. *Pediatr. Nephrol.* **36**, 1439–1446 (2021). 32529323 10.1007/s00467-020-04632-1

[26] Hao, T. K. Prevalence and risk factors for hyponatremia in preterm infants. *Open Access Maced. J. Med. Sci.* **7**, 3201–3204 (2019). 31949516 10.3889/oamjms.2019.558 PMC6953920

[27] Jochum, F. et al. ESPGHAN/ESPEN/ESPR/CSPEN guidelines on pediatric parenteral nutrition: fluid and electrolytes. *Clin. Nutr.* **37**, 2344–2353 (2018). 30064846 10.1016/j.clnu.2018.06.948

[28] Iacobelli, S., Kermorvant-Duchemin, E., Bonsante, F., Lapillonne, A. & Gouyon, J. B. Chloride balance in preterm infants during the first week of life. *Int. J. Pediatr.* **2012**, 931597 (2012). 22505945 10.1155/2012/931597 PMC3312278

[29] Baraton, L. et al. Impact of changes in serum sodium levels on 2-year neurologic outcomes for very preterm neonates. *Pediatrics* **124**, e655–e661 (2009). 19752079 10.1542/peds.2008-3415

[30] Dalton, J., Dechert, R. E. & Sarkar, S. Assessment of association between rapid fluctuations in serum sodium and intraventricular hemorrhage in hypernatremic preterm infants. *Am. J. Perinatol.* **32**, 795–802 (2015). 25545443 10.1055/s-0034-1396691

[31] Spath, C., Sjostrom, E. S., Ahlsson, F., Agren, J. & Domellof, M. Sodium supply influences plasma sodium concentration and the risks of hyper- and hyponatremia in extremely preterm infants. *Pediatr. Res.* **81**, 455–460 (2017). 27935901 10.1038/pr.2016.264

[32] O’Driscoll, D. N., McGovern, M., Greene, C. M. & Molloy, E. J. Gender disparities in preterm neonatal outcomes. *Acta Paediatr.* **107**, 1494–1499 (2018). 10.1111/apa.14390 29750838

[33] Christians, J. K. et al. Sex differences in the effects of prematurity and/or low birthweight on neurodevelopmental outcomes: systematic review and meta-analyses. *Biol. Sex. Differ.* **14**, 47 (2023). 37434174 10.1186/s13293-023-00532-9 PMC10334669

[34] Duburcq, T. et al. Hypertonic sodium lactate improves fluid balance and hemodynamics in porcine endotoxic shock. *Crit. Care* **18**, 467 (2014). 25125153 10.1186/s13054-014-0467-3 PMC4243725

[35] Olofsson, P. Umbilical cord ph, blood gases, and lactate at birth: normal values, interpretation, and clinical utility. *Am. J. Obstet. Gynecol.* **228**, S1222–S1240 (2023). 37164495 10.1016/j.ajog.2022.07.001

[36] Langer, T., Brusatori, S. & Gattinoni, L. Understanding base excess (Be): merits and pitfalls. *Intensive Care Med.* **48**, 1080–1083 (2022). 35639122 10.1007/s00134-022-06748-4 PMC9304040

[37] Severinghaus, J. W. & Astrup, P. B. History of Blood Gas Analysis. II. pH and acid-base balance measurements. *J. Clin. Monit.* **1**, 259–277 (1985). 3913750 10.1007/BF02832819

[38] Massenzi, L. et al. Use of Intravenous sodium bicarbonate in neonatal intensive care units in Italy: a nationwide survey. *Ital. J. Pediatr.* **47**, 63 (2021). 33706798 10.1186/s13052-021-00955-3 PMC7953611

[39] Ali, A., Ong, E. Y., Sadu Singh, B. K. & Cheah, F. C. Comparison between sodium acetate and sodium chloride in parenteral nutrition for very preterm infants on the acid-base status and neonatal outcomes. *Pediatr. Gastroenterol. Hepatol. Nutr.* **23**, 377–387 (2020). 32704498 10.5223/pghn.2020.23.4.377 PMC7354868

[40] Ekblad, H., Kero, P. & Takala, J. Slow sodium acetate infusion in the correction of metabolic acidosis in premature infants. *Am. J. Dis. Child* **139**, 708–710 (1985). 4014095 10.1001/archpedi.1985.02140090070032

[41] Kasik, J. W., Vafai, J. & Goodrich, P. Sodium acetate infusion to correct acidosis in premature infants. *Am. J. Dis. Child* **140**, 9–10 (1986). 3942118

[42] Richards, C. E., Drayton, M., Jenkins, H. & Peters, T. J. Effect of different chloride infusion rates on plasma base excess during neonatal parenteral nutrition. *Acta Paediatr.* **82**, 678–682 (1993). 8374218 10.1111/j.1651-2227.1993.tb18039.x

[43] Peters, O., Ryan, S., Matthew, L., Cheng, K. & Lunn, J. Randomised controlled trial of acetate in preterm neonates receiving parenteral nutrition. *Arch. Dis. Child Fetal Neonatal Ed.* **77**, F12–F15 (1997). 9279176 10.1136/fn.77.1.f12 PMC1720676

[44] Junarsa, N., Somasetia, D. & Sambas, D. Impact of hyperosmolar sodium-lactate resuscitation on lactate clearance in pediatric severe sepsis. *Am. J. Epidemiol. Infect. Dis.* **3**, 76–79 (2015).

[45] Mustafa, I. & Leverve, X. M. Metabolic and hemodynamic effects of hypertonic solutions: sodium-lactate versus sodium chloride infusion in postoperative patients. *Shock* **18**, 306–310 (2002). 12392272 10.1097/00024382-200210000-00003

[46] Ichai, C. et al. Sodium lactate versus mannitol in the treatment of intracranial hypertensive episodes in severe traumatic brain-injured patients. *Intensive Care Med.* **35**, 471–479 (2009). 18807008 10.1007/s00134-008-1283-5

[47] Banker, B. Q. & Larroche, J. C. Periventricular leukomalacia of infancy. A form of neonatal anoxic encephalopathy. *Arch. Neurol.* **7**, 386–410 (1962). 13966380 10.1001/archneur.1962.04210050022004

[48] Wisnowski, J. L. et al. Altered glutamatergic metabolism associated with punctate white matter lesions in preterm infants. *PLoS ONE* **8**, e56880 (2013). 23468888 10.1371/journal.pone.0056880 PMC3582631

[49] Karagiannis, A. et al. Lactate is an energy substrate for rodent cortical neurons and enhances their firing activity. *Elife* **10**, e71424 (2021). 10.7554/eLife.71424 PMC8651295 34766906

[50] Wyss, M. T., Jolivet, R., Buck, A., Magistretti, P. J. & Weber, B. In vivo evidence for lactate as a neuronal energy source. *J. Neurosci.* **31**, 7477–7485 (2011). 21593331 10.1523/JNEUROSCI.0415-11.2011 PMC6622597

[51] Plourde, G. et al. Neuroprotective effects of lactate and ketone bodies in acute brain injury. *J. Cereb. Blood Flow Metab.* **44**, 1078–1088 (2024). 10.1177/0271678X241245486 PMC11179615 38603600

[52] Buscemi, L., Blochet, C., Magistretti, P. J. & Hirt, L. Hydroxycarboxylic acid receptor 1 and neuroprotection in a mouse model of cerebral ischemia-reperfusion. *Front. Physiol.* **12**, 689239 (2021). 34093243 10.3389/fphys.2021.689239 PMC8176103

