# Supplementary material for: Positive impact of sodium L-lactate supplementation on blood acid-base status in preterm newborns
Source: Pediatr Res. 2025 Mar 6;98(2):611–20. doi: 10.1038/s41390-025-03963-9 (PMC12454137; doi:10.1038/s41390-025-03963-9)
Supplement: Supplementary file 1 — PedRes Suppl Fig [file 41390_2025_3963_MOESM1_ESM.pdf]

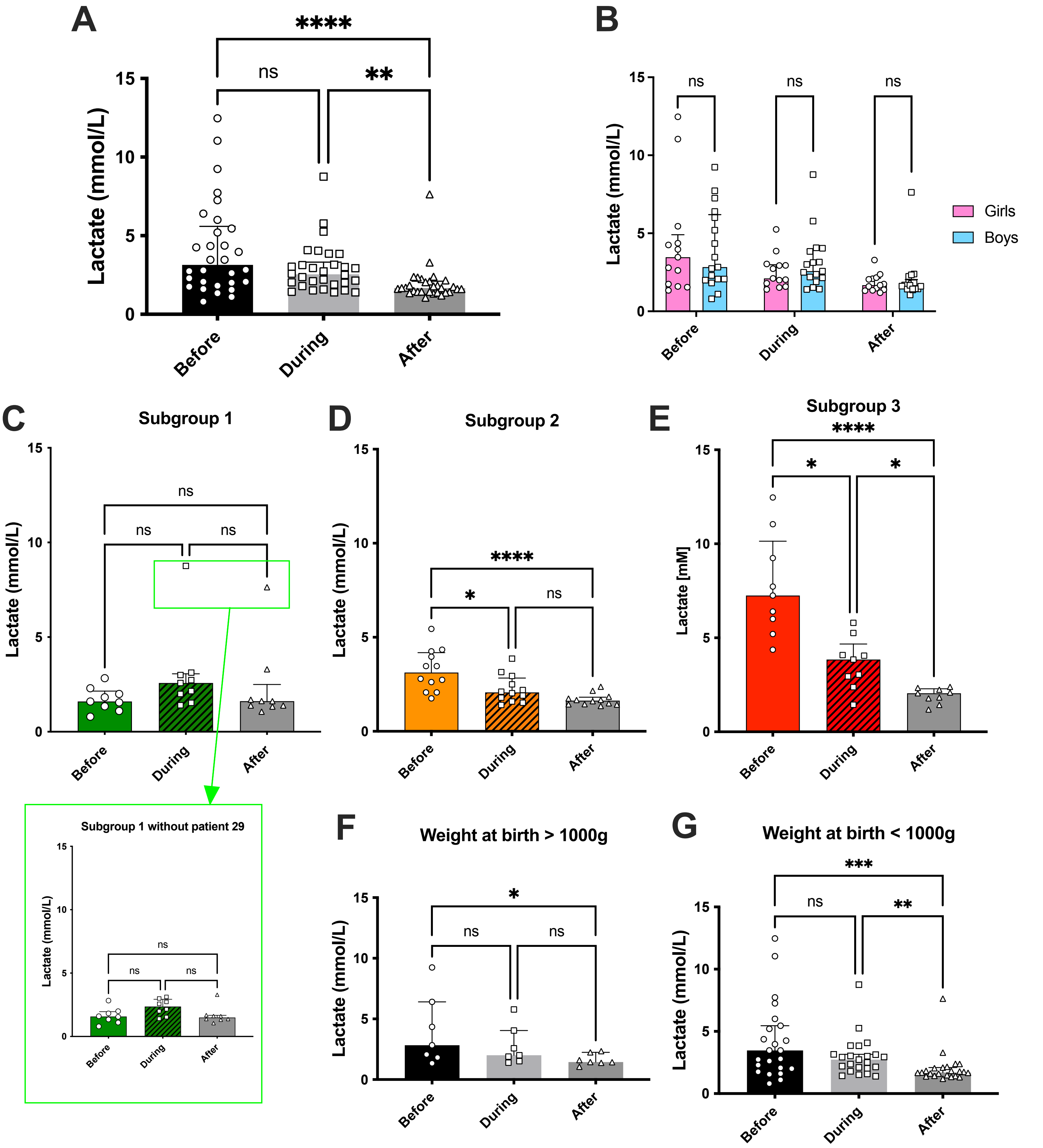

***Supplementary Figure S1:** Evolution of lactatemia before, during and after sodium L-lactate infusion for all preterm newborns who received sodium L-lactate (**A**, n = 30) or according to gender distribution (**B**). **C:** Evolution of lactatemia after birth before, during and after sodium L-lactate infusion for the preterm newborns with low lactatemia at birth (subgroup 1, blood lactate concentration < 2 mmol/L, n = 9). **D:** Evolution of lactatemia after birth before, during and after sodium L-lactate infusion for the preterm newborns with moderate hyperlactatemia at birth (subgroup 2, 2 ≤ lactatemia ≤ 5 mmol/L, n = 12). **E:** Evolution of lactatemia after birth before, during and after sodium L-lactate infusion for the preterm newborns with high lactatemia at birth (subgroup 3, lactatemia > 5 mmol/L, n = 9). *: p < 0.05, **: p < 0.01, and ****: p < 0.0001. ns: non significative. Green square: data from patient number 29, who presented a major increase in lactatemia during and after the sodium L-lactate infusion, concomitantly with a hemodynamic deterioration in a context of septic shock at 3 1/2 days of life, were removed in the green framed graphic. Data are presented as median and interquartile range.*

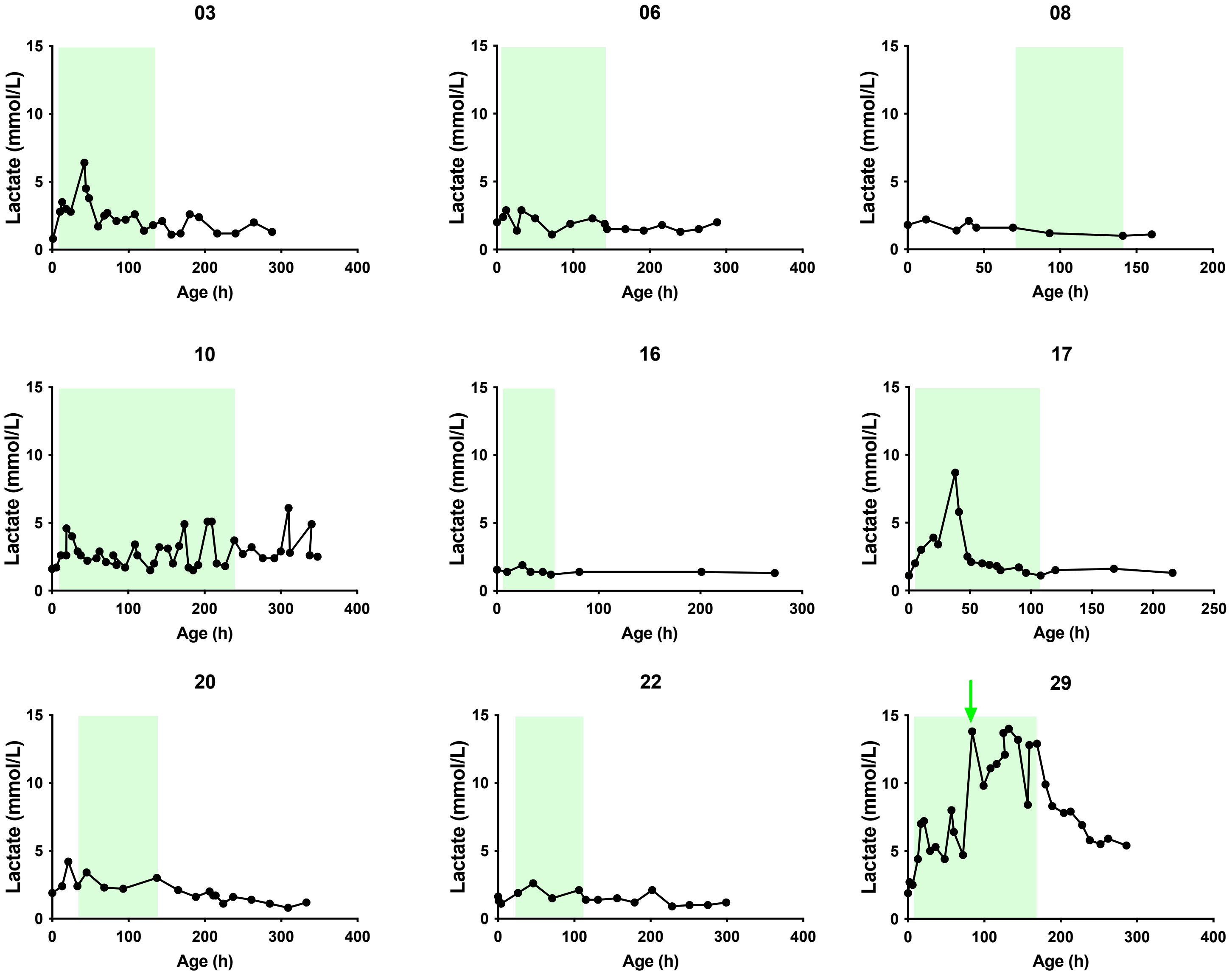

Supplementary Figure S2: *Kinetics of lactatemia for each patient in the subgroup 1 of the Na-Lact group. The colored area corresponds to the time period during which sodium L-lactate was infused. Patient number is indicated above each graph. Green arrow: diagnosis of hemodynamic deterioration and septic shock in patient 29 at 84h of life.*

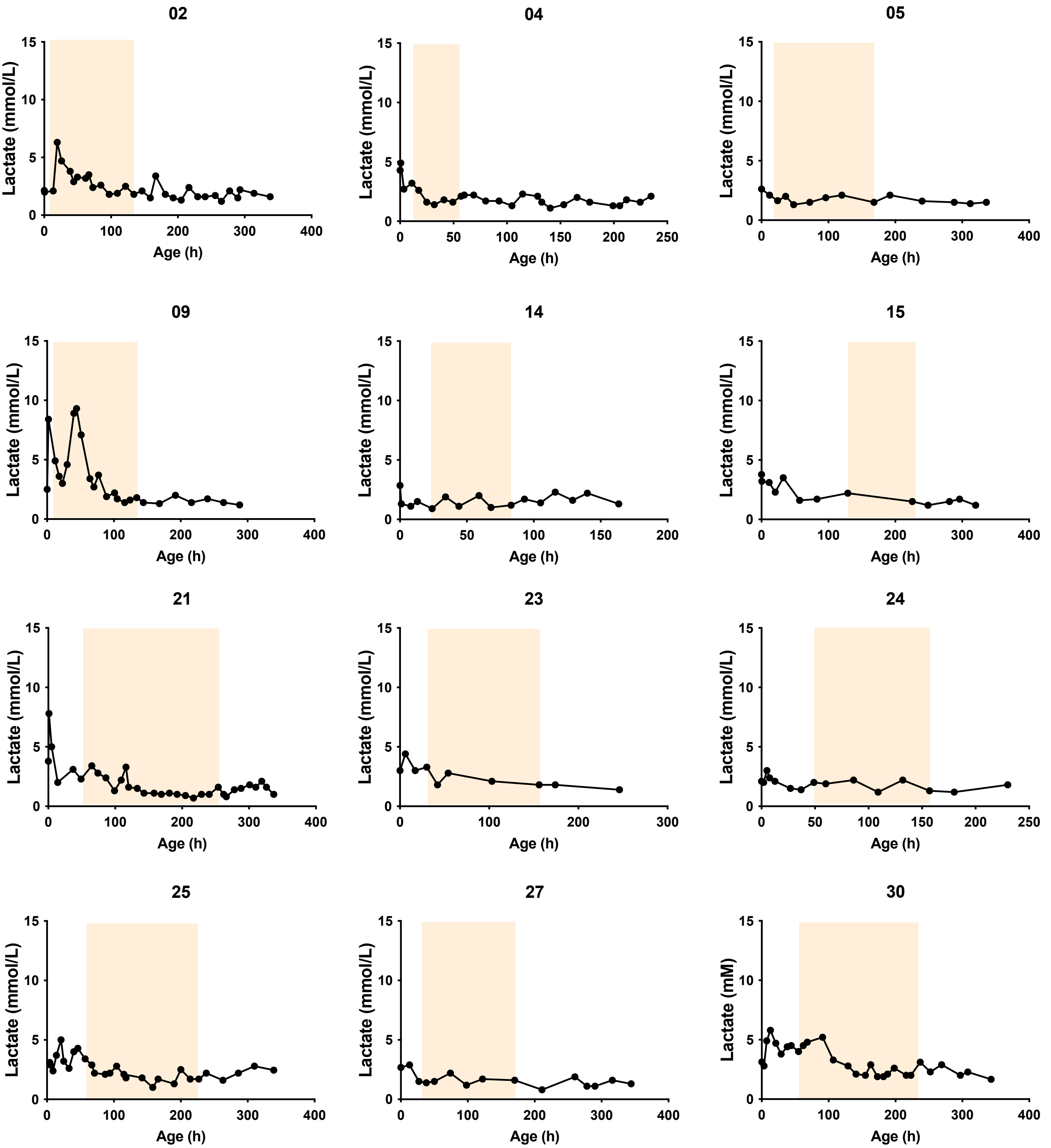

Supplementary Figure S3: *Kinetics of lactatemia for each patient in the subgroup 2 of the Na-Lact group. The colored area corresponds to the time period during which sodium L-lactate was infused. Patient number is indicated above each graph.*

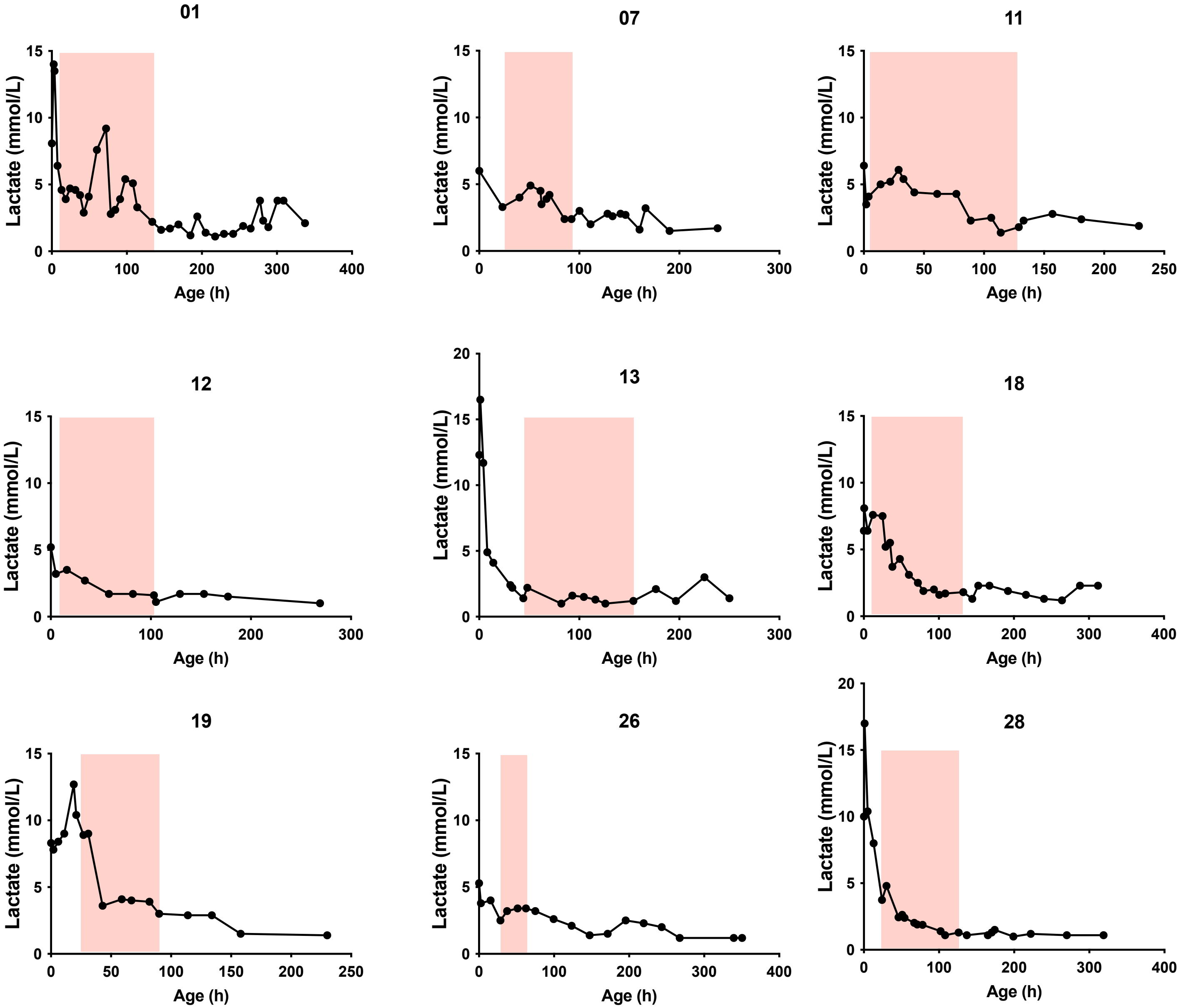

Supplementary Figure S4: *Kinetics of lactatemia for each patient in the subgroup 3 of the Na-Lact group. The colored area corresponds to the time period during which sodium L-lactate was infused. Patient number is indicated above each graph.*

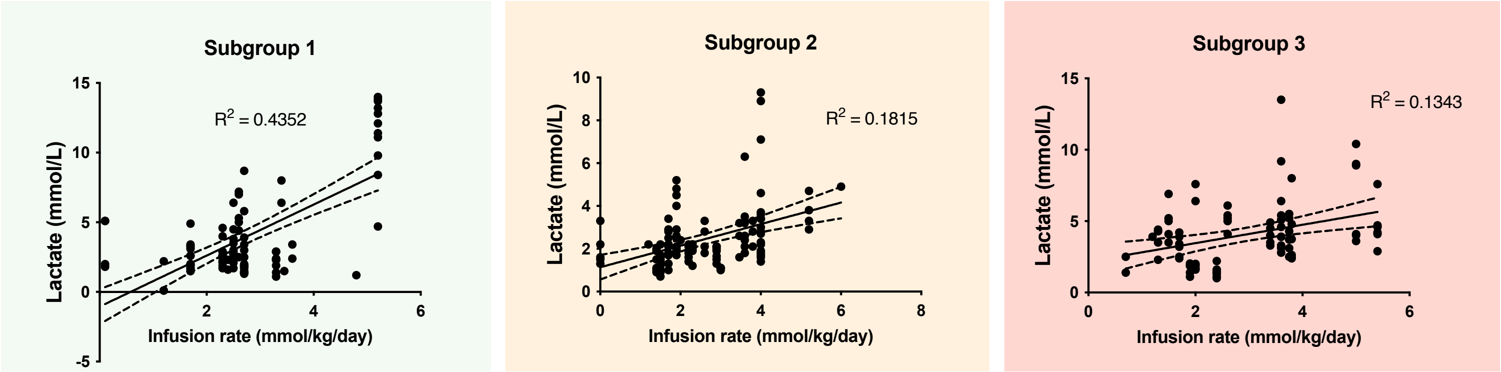

Supplementary Figure S5: *Correlations between blood lactate concentrations (mmol/L) and infusion rates of the sodium L-lactate solution (mmol/kg/day) for each subgroup. Correlation coefficients are indicated on graphs.*

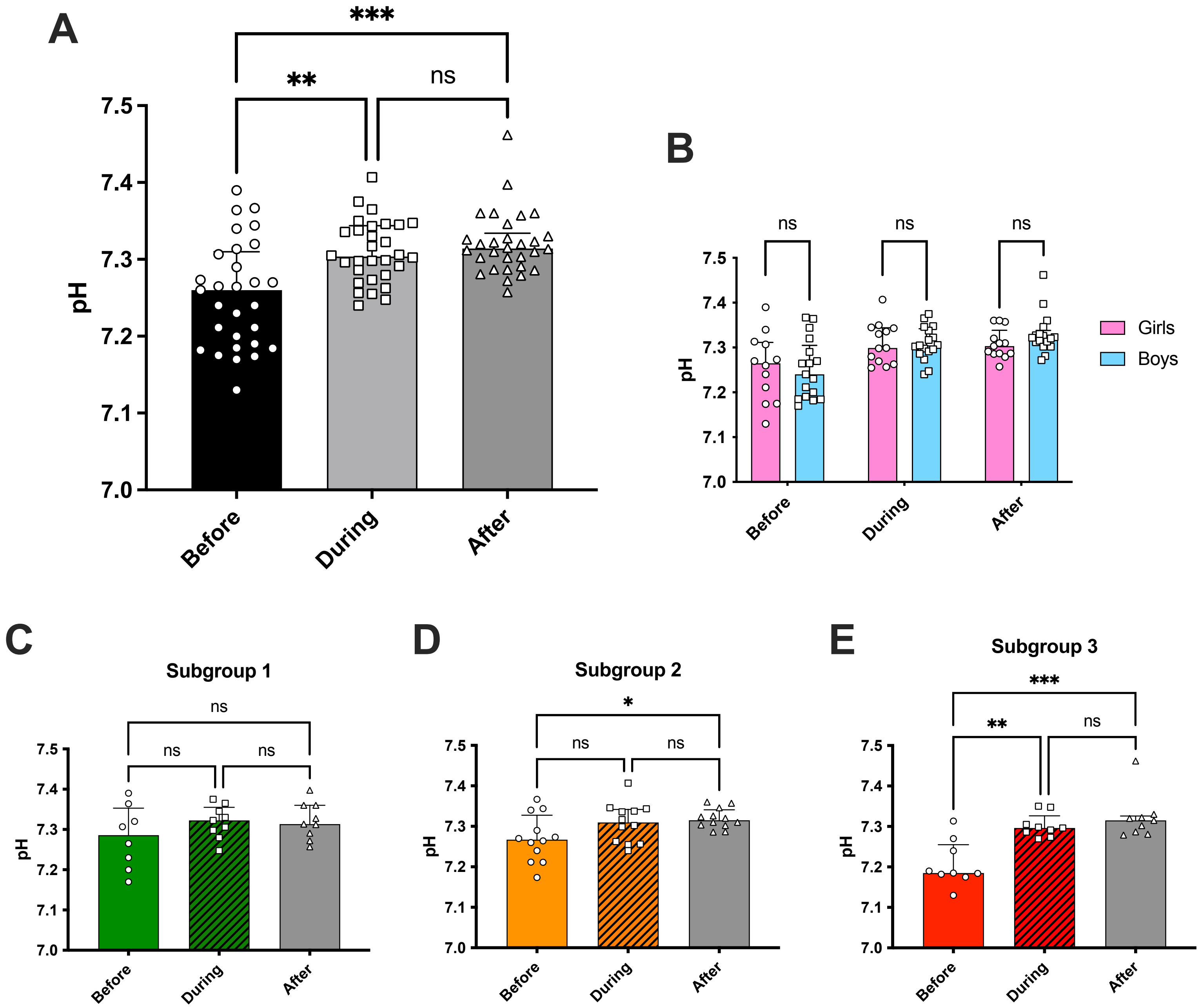

Supplementary Figure S6: *Evolution of blood pH before, during and after sodium L-lactate infusion for all preterm newborns who received sodium L-lactate (**A**, n = 30) or according to gender distribution (**B**). **C:** Evolution of blood pH after birth before, during and after sodium L-lactate infusion for the preterm newborns with low lactatemia at birth (subgroup 1, blood lactate concentration < 2 mmol/L, n = 9). **D:** Evolution of blood pH after birth before, during and after sodium L-lactate infusion for the preterm newborns with moderate hyperlactatemia at birth (subgroup 2, 2 ≤ lactatemia ≤ 5 mmol/L, n = 12). **E:** Evolution of blood pH after birth before, during and after sodium L-lactate infusion for the preterm newborns with high lactatemia at birth (subgroup 3, lactatemia > 5 mmol/L, n = 9). *: p < 0.05, ***: p < 0.001, and ****: p < 0.0001. ns: not significant. Data are presented as median and interquartile range.*

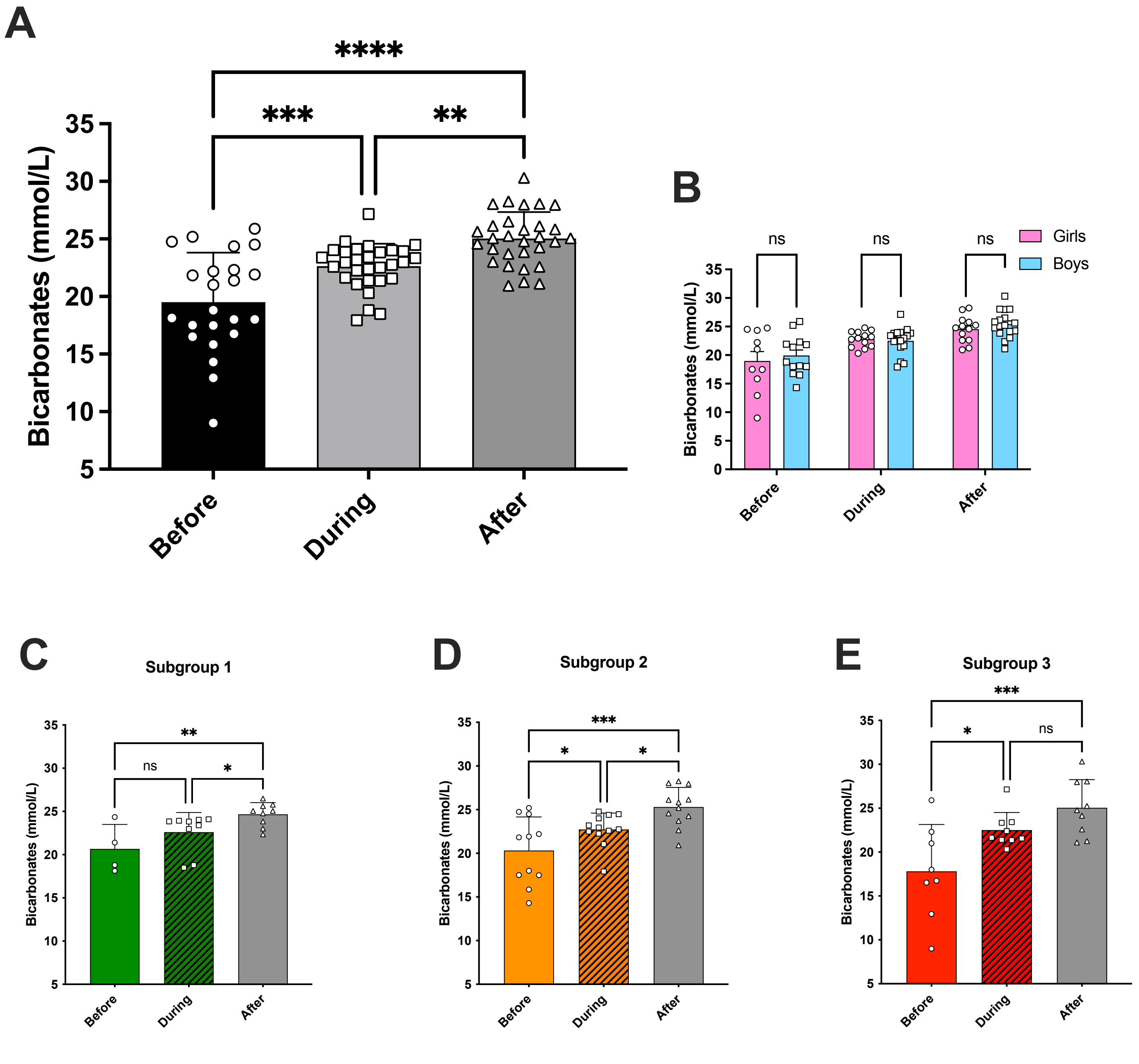

Supplementary Figure S7: *Evolution of bicarbonates before, during and after sodium L-lactate infusion for all preterm newborns who received sodium L-lactate (**A**) or according to gender distribution (**B**).* ***C:*** *Evolution of bicarbonates after birth before, during and after sodium L-lactate infusion in the subgroup 1.* ***D:*** *Evolution of bicarbonates after birth before, during and after sodium L-lactate infusion in the subgroup 2.* ***E:*** *Evolution of bicarbonates after birth before, during and after sodium L-lactate infusion in the subgroup 3. *: $p < 0.05$, **: $p < 0.01$, ***: $p < 0.001$,* and *****: $p < 0.0001$. ns: not significant.*

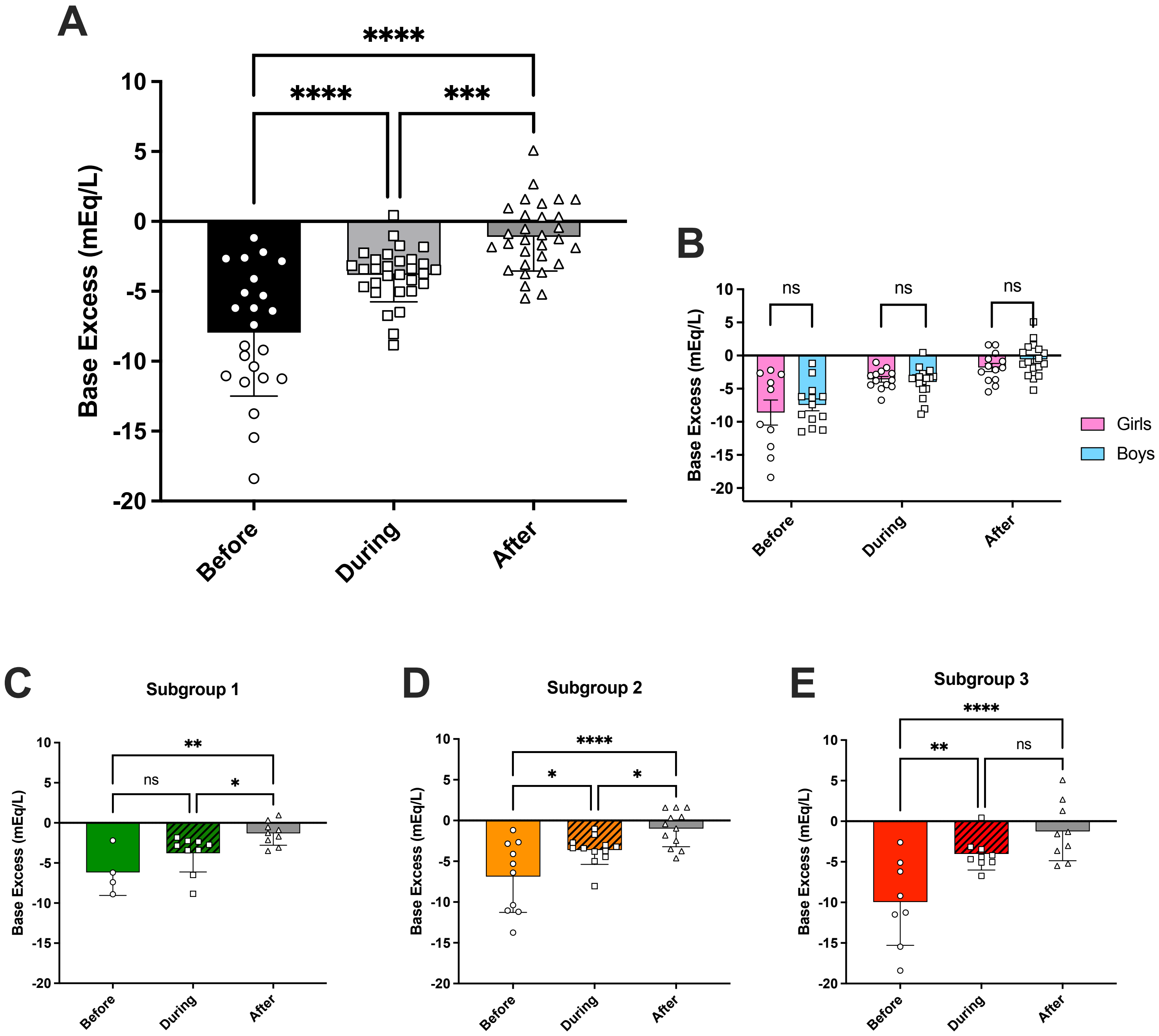

Supplementary Figure S8: *Evolution of base excess before, during and after sodium L-lactate infusion for all preterm newborns who received sodium L-lactate (**A**) or according to gender distribution (**B**). **C:** Evolution of base excess after birth before, during and after sodium L-lactate infusion in the subgroup 1. **D:** Evolution of base excess after birth before, during and after sodium L-lactate infusion in the subgroup 2. **E:** Evolution of base excess after birth before, during and after sodium L-lactate infusion in the subgroup 3. *: p < 0.05, **: p < 0.01, ***: p < 0.001,* and *****: p < 0.0001. ns: not significant.*
